# Polycyclic Aromatic Hydrocarbons in Soil and Vegetation of Niger Delta, Nigeria: Ecological Risk Assessment

**DOI:** 10.1155/2023/8036893

**Published:** 2023-07-20

**Authors:** Esther Amaka Okoye, Anthonet N. Ezejiofor, Ify L. Nwaogazie, Chiara Frazzoli, Orish E. Orisakwe

**Affiliations:** ^1^African Centre of Excellence for Oilfield Chemicals Research (ACE-CEFOR), University of Port Harcourt, PMB, 5323 Port Harcourt, Choba, Nigeria; ^2^African Centre of Excellence for Public Health and Toxicological Research (ACE-PUTOR), Port Harcourt, Nigeria; ^3^Department of Experimental Pharmacology & Toxicology, Faculty of Pharmacy, University of Port Harcourt, PMB, 5323 Port Harcourt, Rivers State, Nigeria; ^4^Department Cardiovascular and Endocrine-Metabolic Diseases and Ageing, Istituto Superiore di Sanità, Rome, Italy

## Abstract

The Niger Delta, Nigeria, is noted for crude oil exploration. Whereas there seems to be a handful of data on soil polycyclic aromatic hydrocarbon (PAH) levels in this area, there is a paucity of studies that have evaluated soil and vegetation PAHs simultaneously. The present study has addressed this information gap. Fresh *Panicum maximum* (Jacq) (guinea grass), *Pennisetum purpureum* Schumach (elephant grass), *Zea mays* (L.) (maize), and soil samples were collected in triplicate from Choba, Khana, Trans-Amadi, Eleme, Uyo, and Yenagoa. PAHs determination was carried out using GC-MS. The percentage composition of the molecular weight distribution of PAHs, the molecular ratio of selected PAHs for identification of possible sources, and the isomeric ratio and total index of soil were evaluated. *Pennisetum purpureum* Schumach (elephant grass) from Uyo has the highest (10.0 mg·kg^−1^) PAH while *Panicum maximum* (Jacq) (guinea grass) has the highest PAH (32.5 mg·kg^−1^ from Khana. *Zea mays* (L.) (maize) from Uyo (46.04%), *Pennisetum purpureum* Schumach (elephant grass) from Trans-Amadi (47.7%), guinea grass from Eleme (49.2%), and elephant grass from Choba (39.9%) contained the highest percentage of high molecular weight (HMW) PAHs. Soil samples from Yenagoa (53.5%) and Khana (55.3%) showed the highest percentage of HMW PAHs. The total index ranged 0.27–12.4 in Uyo, 0.29–8.69 in Choba, 0.02–10.1 in Khana, 0.01–5.53 in Yenagoa, 0.21–9.52 in Eleme, and 0.13–8.96 in Trans-Amadi. The presence of HMW PAHs and molecular diagnostic ratios suggest PAH pollution from pyrogenic and petrogenic sources. Some soils in the Niger Delta show RQ_(NCs)_ values higher than 800 and require remediation to forestall ecohealth consequences.

## 1. Introduction

Polycyclic aromatic hydrocarbons (PAHs) are ubiquitous and persistent organic pollutants consisting of two or more fused rings [1–3]. Their structural stability, semivolatility, and hydrophobicity make them ubiquitous in the environment [4–7]. PAHs have been implicated in diverse toxicities including mutagenicity, teratogenicity, neurotoxicity, genotoxicity, and carcinogenicity in humans [8–12]. Given these public health concerns, 16 PAHs have been recommended for the priority control list by the United States Environmental Protection Agency (USEPA) due to their carcinogenicity, neurotoxicity, and genotoxicity [9]. Furthermore, PAHs are known to migrate and transform among different environmental matrices, including those interfacing food chains, thus affecting both animal and human health [13].

The environmental persistence and public health importance of PAHs in recent years have attracted global attention [14–16]. The principal sources of PAHs in different environmental matrices (soil, water, atmosphere, and food) are natural sources and anthropogenic processes including diverse industrial activities, especially those involving incomplete combustion of coal. Similarly, crude oil and petroleum manufacturing process also produce significant amounts of PAHs [17–19].

Although the uptake of PAH by leaves is mainly by gaseous deposition [20], some reports have demonstrated that leaves can accumulate PAHs from contaminated soils through their roots [21–23]. Nevertheless, Su and Zhu [24] suggested that the PAHs transported from roots to shoots may be negligible, with other researchers affirming that atmospheric PAHs are dominant contributors to the total PAHs in leaves [25, 26].

Long periods of crude oil exploitation in many countries have led to complex contamination by petroleum hydrocarbons and PAHs in many cities. For instance, some studies from China have reported an average content of 16 PAHs (∑_16_ PAHs) of 1840 *μ*g/kg in Daqing street dust [27]. All in all, it is known that the pollution effects of PAHs on ecological land in oil- producing cities and surrounding communities have great significance on urban ecological security and environmental health [28, 29]. The Niger Delta area of Nigeria is one of the major crude oil-exploring regions in the world with inundated cases of oil spills, aerial deposition of organic by-products originating from flared gases and massive environmental degradation by both inorganic and organic pollutants like PAHs [10]. Some researchers have reported concentrations and compositional patterns of PAHs that can be employed in understanding the effects, sources, fate, and transport of PAHs in soils, as well as environmental quality management in the Niger Delta, Nigeria [30, 31]. PAHs strongly accumulate in the food chain and are subsequently transferred to humans, thereby posing a threat to human health [32, 33]. Some skeletal surveys of PAHs in agricultural soils have been carried out in Niger Delta [34–36]. Some of these surveys suggest that some agricultural soils in the Niger Delta, Nigeria, suffer from PAH pollution due mainly to point source pollution. Surface and underground water are polluted with PAHs in Nigeria [37, 38]. PAHs can transfer from soil to borehole water [39]. Although some studies have reported soil and vegetation PAH contamination, information remains sparse on PAH contamination of both soils and vegetation from the same location [40]. Understanding of the spatial distribution of PAHs in agricultural topsoil is critical for environmental management and the safety of agricultural produce.

As recently discussed [41], the One Health strategy, including environmental health and food safety, can help risk assessors and risk managers in prioritising actions for the prevention and mitigation of PAH pollution and its spread and accumulation. In the present study, we evaluated the whole ecological risk of PAHs in soil and vegetation samples from farmlands in six major cities of Niger Delta, Nigeria.

## 2. Materials and Methods

### 2.1. Study Area

The Niger Delta, Nigeria, is the third largest mangrove forest and the second largest delta in the world. It falls within the central coastlands of southern Nigeria [10]. The Niger Delta area is well known for crude oil exploration and environmental pollution. According to Okoye et al. [10], the black to greyish brown and dark grey soils are acidic with slight to moderate electrical conductivity and high organic carbon content.

### 2.2. Sampling and Sample Treatment

#### 2.2.1. Detailed Sampling Locations (GPS)

**Figure 1:**
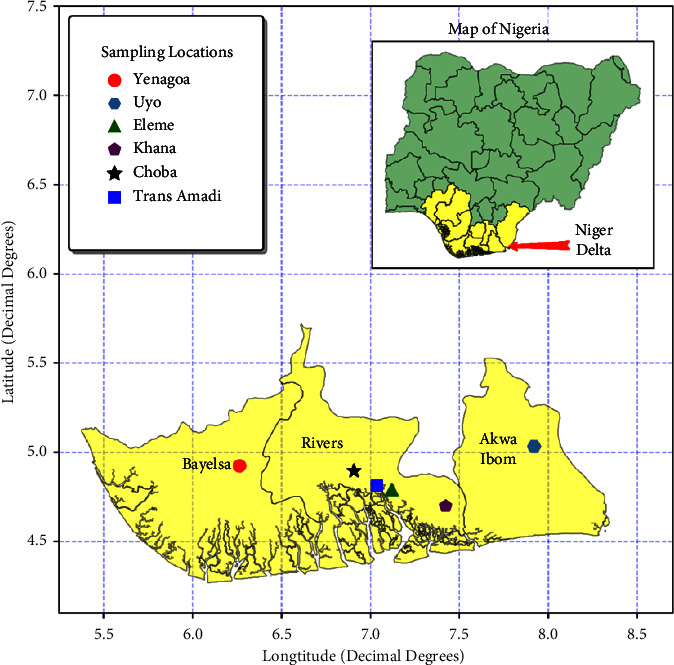
Map of sampling location in the Niger Delta Region, reproduced from the study by Okoye et al., 2022. Heavy metals and arsenic in soil and vegetation of Niger Delta, Nigeria: ecological risk assessment. Case studies in chemical and environmental engineering, 6, p.100222.

Soil and fresh plant (guinea grass, elephant grass, and maize) samples were sampled in Choba-4°53′16.6″N 6°54′31.3″E, Khana-4°39′36.0″N 7°22′39.4″E, Trans-Amadi-4°48′53″N 7°2′14″E, Eleme-4°47′24.2″N 7°07′47.6″E, Uyo-5°01′59.0″N 7°56′15.8″E, and Yenegoa-4°55′29″N 6°15′51″E in the Niger Delta.  Figure 1 shows the sampling sites. The sampling, done in triplicate, was carried out during the period of mass flowering of plants, specifically in January 2018. Soil samples were sampled from 0 to 5 cm depth as the most root-inhabited soil layer according to the US EPA Method 610 (U.S. EPA, 1977). The sampled soil was cleaned of plant residues and other inclusions, ground in a porcelain mortar, and passed through a sieve with a hole diameter of 1 mm. Plants were dried and ground up to a hole diameter of 1 mm for analytical analysis.

### 2.3. Analytical Determinations

#### 2.3.1. Information on LOD, LOQ, and Calibration (QA/QC)

Analyses of PAHs were done using gas chromatography (6890 series and 6890 plus) equipped with a dual detector (FID-ECD), dual column, TriPlus AS autosampler with helium carrier gas, and a quadrupole mass spectrometer (Agilent 5975 MSD) based on the US EPA method 8100. This analytical procedure has been described previously [42–45]. Briefly, the extraction of PAHs from the samples was done with a sonicator (ultrasonic bath, Elmsonic S40H) in accordance with US SW-846 Method 3550. Two grams of either soil or plant samples were extracted with a 50 : 50 mixture of acetone and methylene chloride (analytical grade), spiked with 1 ml of PAH internal standard, and shaken thoroughly for proper mixing before being placed in an ultrasonic bath. Thereafter, 2.00 *μ*l of each sample extracts were injected into the GC port set at column conditions: HP-5cross-linked PH-ME siloxane, length of 30 m, I.D: 0.25 mm, thickness of 1 *μ*m with helium carrier gas set in the spitless, constant flow mode with a 1.2 ml/min flow rate. Other GC and MS operating setups were done according to the instrument's method of development as specified in the operating instruction manual. Identification and quantification of individual PAHs were based on an internal calibration standard containing known concentrations of the 16 PAHs [43–45]. The specificity of the 16 PAHs sought in the samples was confirmed by the presence of transition ions (quantifier and qualifier) as shown by their retention times which corresponded to those of their respective standards. The measured peak area ratios of precursor to quantifier ions were in close agreement with those of the standards. The 16 PAHs analyzed were the following: napthalene (Nap) CAS No. 91-20-3, acenaphthylene (Acy) CAS No. 208-96-8, acenapthene (Ace) CAS No. 83-32-9, fluorene (Flu) CAS No. 86-73-7, phenanthrene (Phe) CAS No. 85-01-8, anthracene (Ant) CAS No. 120-12-7, fluoranthene (Flt) CAS No. 206-44-0, pyrene (Pyr) CAS No. 129-00-0, benzo[*a*]anthracene (BaA) CAS No. 56-55-3, chrysene (Cry) CAS No. 218-01-9, benzo[*b*]fluoranthene (BbF) CAS No. 205-99-2, benzo[*k*]fluoranthene (BkF) CAS No. 207-08-9, benzo[*a*]pyrene (BaP) CAS No. 50-32-8, dibenzo[*ah*]anthracene (DahA) CAS No. 53-70-3, benzo[*ghi*]perylene (BghiP) CAS No. 191-24-2, and indeno[1,2,3-*cd*]pyrene (Ind) CAS No. 193-39-5. The CAS No. is a unique identification number assigned by the Chemical Abstracts Service (CAS), US, to every chemical substance described in the open scientific literature.

The detection limit (LOD) is estimated as three times the background noise (IUPAC criterion). The blank samples remained always below the quantification limit (LOQ). Table S1 shows the reproducibility relative standard deviation (RSDr; *n* = 6), repeatability relative standard deviation (RSDr; *n* = 6), recoveries, linear range, LOQ, LOD, and coefficient of estimation (*r*^2^).

### 2.4. Data Analysis

SPSS version 17.0 software (SPSS Inc., USA) was used to perform all statistical analyses. The data were analyzed to test for the significance of observed differences in the PAH content by using analysis of variance (ANOVA), while the Tukey test was used to establish if the observed differences in the mean content of PAHs from the different cities were significant.

The sources of the soil PAHs from different cities were evaluated by using PAH isomeric ratios.

The risk quotient in ecological risk assessment is defined as the level of risk produced by a particular PAH and is estimated from the risk quotient (RQ), as shown in the following equation:

(1) $$\begin{array}{c} RQ=\frac{C_{PAHs}}{C_{QV}}\text{,} \end{array}$$

where *C*_PAHs_ is the concentration of certain PAHs in the soil and *C*_QV_ is the corresponding quality value concentration for these PAHs in the soil. Cao et al. [46] model was adopted in order to obtain the quality value concentrations in the present study.

The negligible concentrations (NCs) and the maximum permissible concentrations (MPCs) of PAHs are two quality values employed here with corresponding risk quotients, RQ_NCs_ and RQ_MPCs_, respectively, computed from

(2) $$\begin{array}{c} {RQ}_{NCs}=\frac{C_{PAHs}}{C_{QV\left(NCs\right)}}\text{,} \end{array}$$

(3) $$\begin{array}{c} {RQ}_{MPCs}=\frac{C_{PAHs}}{C_{QV\left(MPCs\right)}}\text{,} \end{array}$$

where *C*_QV(NCs)_ is the quality value of the NCs and *C*_QV(MPCs)_ is the quality value of the MPCs of the PAHs in the soil.

The risk caused by a combination of all 16 PAHs can be appraised by calculating RQ∑PAHs_(NCs)_ and RQ∑PAHs_(MPCs)_ in which the values of RQ_NCs_ and RQ_MPCs_ of the individual PAHs that are not less than one are summed, as shown as follows:

(4) $$\begin{array}{c} \overset{RQ}{\sum }{PAHs}_{\left(NCs\right)}=\overset{16}{\underset{i=1}{\sum }}{RQ}_{i\left(NCs\right)}\text{,} \end{array}$$

where RQ_*i*(NCs)_ ≥ 1.

(5) $$\begin{array}{c} \overset{RQ}{\sum }{PAHs}_{\left(MPCs\right)}=\overset{16}{\underset{i=1}{\sum }}{RQ}_{i\left(MPCs\right)}\text{,} \end{array}$$

where RQ_*i*(MPCs)_ ≥ 1.

The RQ denotes as follows: RQ_NCs_ <1.0 indicates that the individual PAH compounds are probably of negligible concern, while RQ_MPCs_ ≥ 1 suggests severe contamination by the individual PAH compound that requires remediation. RQ_NCs_ ≥ 1.0 and RQ_MPCs_ <1 indicate moderate risk posed by a single PAH compound that might require some control and remediation. However, ∑^RQ^PAHs_(*NCs*)_ ≥ 800 and ∑^RQ^PAHs_(MPCs)_=0 imply moderate risk 1. ∑^RQ^PAHs_(NCs)_ < 800 and ∑^RQ^PAHs_(MPCs)_ ≥ 1 indicate that the PAHs constitute a moderate risk 2; ∑^RQ^PAHs_(NCs)_ ≥ 800 and ∑^RQ^PAHs_(MPCs)_ ≥ 1 show a high risk of the ∑16 PAHs in the ecosystem.

## 3. Results and Discussion

### 3.1. Level of PAHs

Table 1 shows the levels of PAHs in soil and elephant grass, maize, and guinea grass samples from Uyo, Choba, Khana, Yenagoa, Eleme, and Trans-Amadi. Guinea grass from Khana had the highest level of total PAH 32.5, whereas elephant grass from Uyo had the highest level of PAH 10.0. Maize samples from Yenagoa showed the highest level of PAH (9.73). Soil samples from Uyo had the highest soil total PAH (8.80).

### 3.2. Source Apportionment

**Figure 2:**
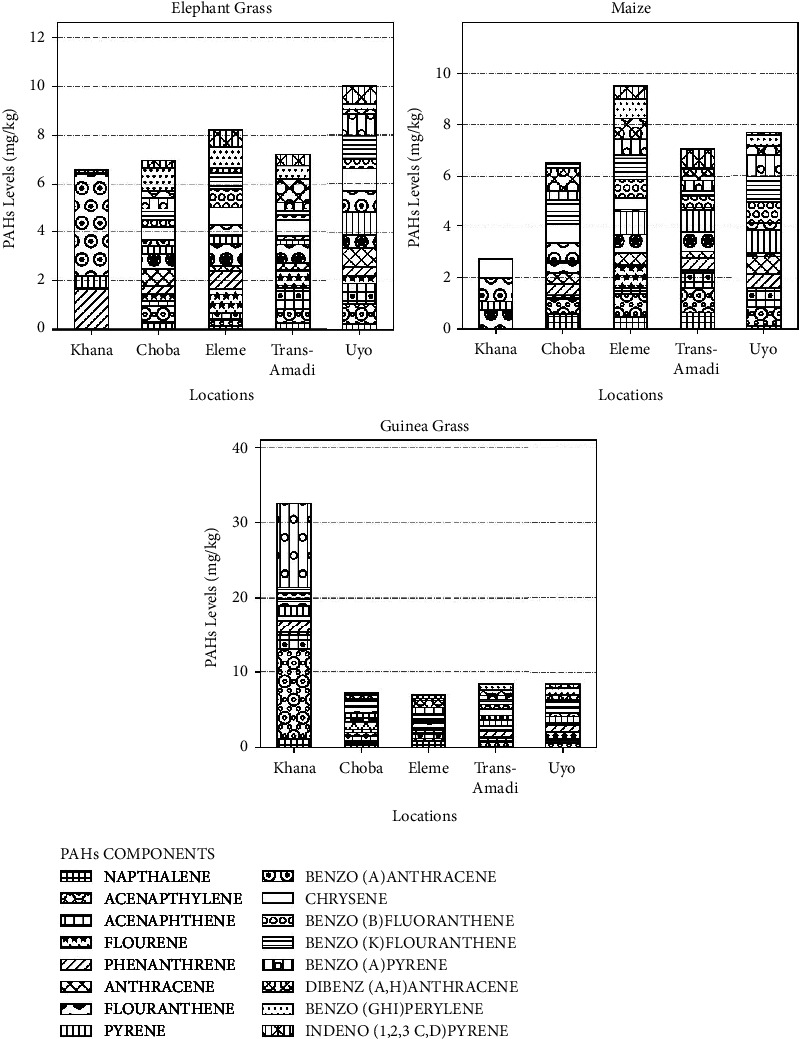
PAHs content in elephant grass (a), maize (b), and guinea grass (c) from different locations in the Niger Delta, mg·kg^−1^.

**Figure 3:**
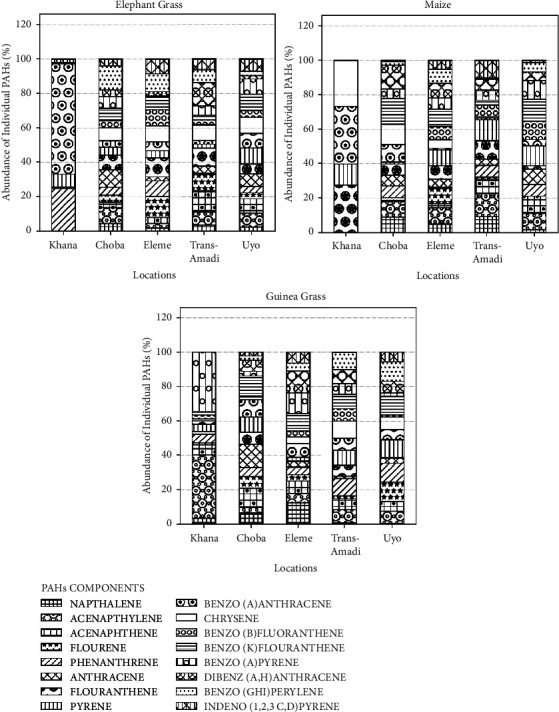
Abundance of individual PAHs (%) in elephant grass (a), maize (b), and guinea grass (c) from different locations in the Niger Delta. Abundance is the percentage of the individual PAH components in the total PAH.

Figures 2 and 3 show the PAHs content in elephant grass 2(a), maize 2(b), and guinea grass 2(c) from different locations in the Niger Delta and the abundance of individual PAHs in elephant grass 3(a), maize 3(b), and guinea grass 3(c) from Choba, Eleme, Khana, Trans-Amadi, Uyo, and Yenagoa in the Niger Delta, respectively.

**Figure 4:**
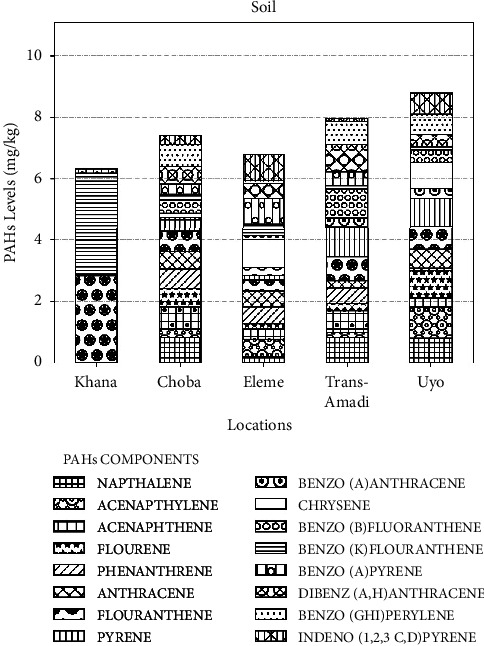
PAH content in soil samples from different locations in the Niger Delta, mg·kg^−1^.

**Figure 5:**
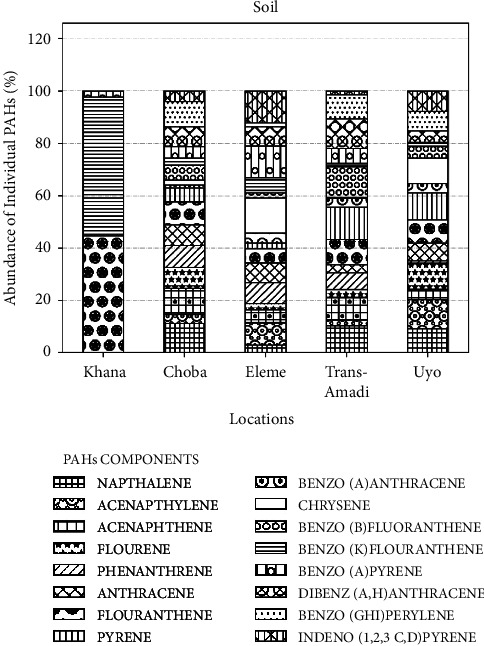
Abundance of individual PAHs (%) in soil samples from different locations in the Niger Delta.

The levels of different PAH content in elephant grass, maize, guinea grass, and soil samples, respectively, from Choba, Eleme, Khana, Trans-Amadi, Uyo, and Yenagoa in the Niger Delta, Nigeria, are provided in Figures 2–5.  Figure 2(a) shows that elephant grass from Uyo had the highest number and content of PAHs whereas Khana had the least number of PAHs and the highest content of BaA (4.22 mg·kg^−1^). The highest content of benzo[*a*]anthracene (BaA) in elephant grass and dibenzo[*ah*]anthracene (DahA) content in guinea grass seen in Khana may be an indication of the severity of PAH pollution, and its impact on ecohealth should be further investigated.

Figure 2(b) shows that the maize from Yenagoa and Eleme had a higher content of different PAHs than Khana which contained a fewer number of PAHs with BaA being the highest. The lowest levels of PAHs were seen in guinea grass 2C from Yenagoa, Eleme, Choba, Trans-Amadi, and Uyo. Guinea grass from Khana, Niger Delta, contain all the PAHs and highest levels of Acy (11.9 mg·kg^−1^) and BaP (11.1 mg·kg^−1^) in comparison to other locations in this study (Figure 2(c)).

The PAHs content in soil samples from different locations in the Niger Delta, and the abundance of individual PAHs in soil samples from different locations in the Niger Delta are shown in Figures 4 and 5, respectively. According to Figures 4 and 5, Khana had the least number of PAHs and the highest concentration of B(k)F and Flt in comparison to other locations. The soil sample from Uyo had the highest number and concentration of PAHs.

A comparison of the total soil PAH levels (mean and ranges) from different countries and present study is shown in Table 2.

The PAH concentrations of agricultural soils from Choba, Eleme, Khana, Trans-Amadi, Uyo, and Yenagoa, Niger Delta, were 7417, 6787, 6325, 7972, 8799, and 7394 *μ*g·kg^−1^, respectively. These PAH levels were higher than most of the soil PAH levels from other countries [61, 69, 70].

**Figure 6:**
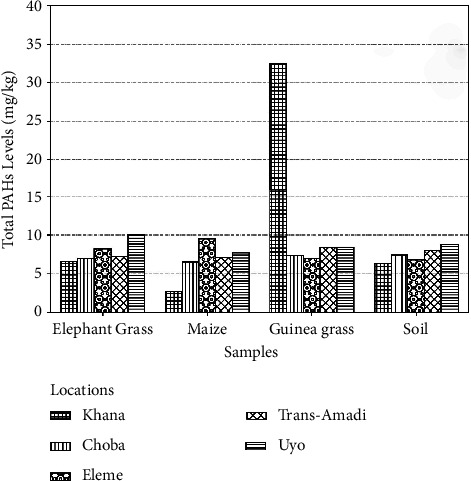
Total PAHs in samples from different locations in the Niger Delta.

Figure 6 shows the total PAHs for the different samples from different locations in the Niger Delta of which Uyo has the highest (10.016 mg·kg^−1^) in elephant grass and Khana has the highest PAH (32.508 mg·kg^−1^) in guinea grass and the least in all the other samples. Vegetal levels (2.698 maize-32.508 mg·kg^−1^ elephant grass) of PAHs were higher than soil sample levels (6.325–8.799 mg·kg^−1^).

**Figure 7:**
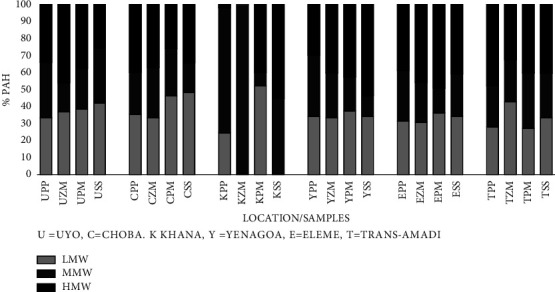
Percentage composition and molecular weight distribution of PAHs in soil and plants from different locations in the Niger Delta. PP = *Pennisetum purpureum* Schumach (elephant grass), ZM = *Zea mays* (L.) (maize), PM = *Panicum maximum* (Jacq) (guinea grass), and SS = soil sample.

Based on the number of rings or molecular structure, the priority PAHs are classified as follows: low molecular weight (LMW) PAHs, i.e., Naph, Acy, Acen, Flu, Phen, and Anth (containing two and three rings), medium molecular weight (MMW) PAHs, i.e., Flan, Pyr, Chry, and BaA (with four rings), and high molecular weight (HMW) PAHs, i.e., BbF, BkF, BaP, IP, DBahA, and BghiP (with five and six rings) [71]. The percentage composition and molecular weight distribution of PAHs in vegetation, i.e., elephant grass, maize, and guinea grass and soils from Uyo, Choba, Khana, Yenagoa, Eleme, and Trans-Amadi in the Niger Delta, Nigeria, are shown in Table 3 and Figure 7. PAHs were grouped in three classes of LMW, MMW, and HMW. Maize from Uyo (46.0%), elephant grass from Trans-Amadi (47.9%), guinea grass from Eleme (49.2%), and elephant grass from Choba (39.9%) contained the highest percentage of HMW PAHs. Soil samples from Yenagoa (53.5%) show the highest percentage of HMW PAHs. Figure 7 shows the different distribution of PAHs according to their molecular weight: for Uyo, maize has more HMW while in guinea grass, elephant grass, and soil samples, LMW was more abundant. For Choba, samples of elephant grass and maize contain more HMW while guinea grass and soil samples have more LMW. For Khana, elephant grass and maize have more MMW PAH and HMW PAH in soil samples. The same trend follows in Yenagoa, Eleme, and Trans-Amadi with HMW in soil samples, guinea grass, and elephant grass, respectively.

The existence of different homologs of PAHs (PAHs with a number of aromatic rings) and PAHs with different molecular weights in the environment suggests their likely origin or sources [72–77].

Although there were higher levels of HMW PAHs in plant/vegetation samples in some cities in the present study, the fairly appreciable presence of 4-ring PAHs and 3-ring PAHs (Table 3 and Figure 7) is indicative of mixed pyrogenic sources. The LMW and MMW PAHs are known to exist both in the vapor and particulate phases [77] and usually reside within the locality of the origin or source. The observation from an additive standpoint that 3-4-rings PAHs predominated in this study suggests localized mixed sources coupled with atmospheric transport [40, 70].

PAHs with less than 4 aromatic rings (LMW PAHs) are typified by grass and industrial oil, wood combustion, and petroleum products (Liu et al. 2017), whereas PAHs with more than 4 aromatic rings (HMW PAHs) signify pyrogenic activities at high temperature including coal combustion and vehicular emissions [78]. The higher levels of HMW PAHs in maize from Uyo, elephant grass from Trans-Amadi, guinea grass from Eleme, elephant grass from Choba, and soil samples from Yenagoa and Khana may implicate vehicular traffic.

Since HMW PAHs tend to reside in closer proximity to emission sources and LMW PAHs are carried to areas far from the emission sources [40, 70], the homolog pattern of PAHs in this study with different molecular weights may be characterized by local combustion sources in addition to atmospheric transported depositions [77, 79].

In addition to using different homologs of PAHs (PAHs with the same number of aromatic rings) and PAHs with different molecular weights, molecular diagnostic ratios of selected PAHs concentrations, including Fle/(Fle + Pyr), Ant/(Ant + Phe), Flt/(Flt + Pyr) in PAH identification, BaA/(BaA + Cry), BbF/BkF, BaP/BghiP, BaP/(BaP + Cry), and Ind/(Ind + BghiP) were also employed as inferential tools in the characterization of possible sources of PAHs from plant and soils samples from Uyo, Choba, Khana, Yenagoa, Eleme, and Trans-Amadi (Table 4). The average ratio of PAHs in plants and soil suggested diverse pyrogenic sources of PAHs emission, such as Fle/(Fle + Pyr) (0.60 (0.59–0.97)) for petrol and diesel [72, 80], Ant/(Ant + Phe) (0.88 (0.61–0.70)) for petroleum and biomass combustions [81], Flt/(Flt + Pyr) (0.45 (0.77–1.67)) for biomass, coal combustion (39), BaA/(BaA + Cry) (0.27 (0.32–1.17)) for petrogenic and combustion of petroleum and biomass [80], BbF/BkF (1.16 (1.27−1.09)) for diesel engine and vehicular emissions [82], BaP/BghiP (0.76 (1.49–1.94)) for vehicular emissions and coal combustions [83], BaP/(BaP + Cry) (0.009 (0.01–0.86)) for gasoline [84], and Ind/(Ind + BghiP) (0.45 (1.30–2.91)) for petrogenic and petroleum combustions (Table 4). Molecular diagnostic ratio analysis suggests disparate combustion activities including petrol, diesel, gasoline, biomass, and coal combustions and vehicular emissions, as the principal sources. Petrogenic sources are also related to automobile workshops and accidental spillage.

The isomeric ratios, namely, Flt/(Flt + Pyr), Ant/(Ant + Phen), Phen/Ant, LMW/HMW, BaA/(BaA + Chry), and IndP/(IndP + BghiP), and the total index of soil from Uyo, Choba, Khana, Yenagoa, Eleme, and Trans-Amadi, Niger Delta, Nigeria, are shown in Table 5. Furthermore, total indexes [85–87] defined as the sum of single indices (aforementioned parameters previously discussed), respectively, were calculated and normalized for the limit value (low temperature sources–high temperature sources) [80].

(6) $$\begin{array}{c} Total\;index=\frac{Ant}{\left(Ant+Phen\right)/01}+\frac{Flt}{\left(Flt+Pyr\right)/0.4}+\frac{BaA}{BaA}+\frac{\text{Chry}}{0.2}+\frac{IndP}{\left(IndP+BghiP\right)/0.5}\text{.} \end{array}$$

Usually, PAHs associated with combustion or high-temperature processes possess a total index that is greater than 4, whereas PAHs originating from petroleum products or low-temperature processes have a total index that is less than 4. In the present study, the total index ranged 0.27–12.4 in Uyo, 0.29–8.67 in Choba, 0.00–10.1 in Khana, 0.01–5.53 in Yenagoa, 0.21–9.52 in Eleme, and 0.13–8.96 in Trans-Amadi. All the sampling locations had total index values greater than 4. It can therefore be inferred from the total index values that PAHs emanated from low- and high-temperature combustion processes. These observations seem to be in agreement with previous results from Warri [46].

### 3.3. Ecological Risk Assessment

Table 6 shows the RQ_(NCs)_ and RQ∑PAHs_(NCs)_ and RQ_(MPCs)_ and RQ∑PAHs_(MPCs)_ for the 16 priority PAHs in soils from Uyo, Choba, Khana, Yenagoa, Eleme, and Trans-Amadi, Niger Delta, Nigeria. RQ∑PAHs_(NCs)_ values less than 800 are indicative of low ecological risk of PAHs while RQ∑PAHs_(NCs)_ higher than 800 connote higher ecological risk of PAHs. Although some soil samples from the various sampling locations had RQ_(NCs)_ values greater than 800 in the present study, preponderance of the samples showed RQ_(NCs)_ values less than 800 which is suggestive of a low ecological risk of PAHs in these soils. Some previous studies in Nigeria have reported similar RQ_(NCs)_ values less than 800 [88]. The RQ∑PAHs_(MPCs)_ values were greater than 1 except for soil samples from Khana. In this study, Nap, Acy, Ace, Flu, Ant, Flt, and Pyr were the major causes of the ecological risk of PAHs in soils from Uyo, Choba, Yenagoa, Eleme, and Trans-Amadi, Niger Delta, Nigeria. The ecosystem risk of PAHs in soils from Khana was majorly, whereas due to Flt, Phe contributed less to the ecosystem risk of PAHs in soils from Uyo. Similarly, Ant contributed less to the ecosystem risk of PAHs in soils from Choba, Yenagoa, Eleme, and Trans-Amadi, Niger Delta, Nigeria.

## 4. Conclusion

Nap, Acy, Ace, Flu, Ant, Flt, and Pyr were the major causes of the ecological risk due to PAHs in soils from Uyo, Choba, Yenagoa, Eleme, and Trans-Amadi in the Niger Delta, Nigeria. The ecological risk assessment of PAHs derived from isomeric ratios suggested that the PAHs in soils and vegetation samples from Choba, Yenagoa, Eleme, and Trans-Amadi emanated from pyrogenic processes including traffic emissions, fossil fuels, and biomass combustion as well as petrogenic sources such as occasional spills of liquid petroleum fuels and discharges from automobile workshops.

From the risk quotient standpoint, some soil samples from the Niger Delta had RQ_(NCs)_ values greater than 800, thus indicating that soil may require remediation to forestall ecohealth consequences.

## Figures and Tables

**Table 1:** Concentration of PAHs in soil and plant samples from different locations in the Niger Delta.

Samples	Nap	Acy	Ace	Flu	Phe	Ant	Flt	Pyr	BaA	Cry	BbF	BkF	BaP	DahA	BghiP	Ind	Total
*Uyo*																	
Elephant grass	0.25	0.79	0.85	0.35	0.36	0.74	0.57	0.92	0.85	0.93	0.40	0.96	0.89	0.18	0.25	0.72	10.0
Maize	0.14	0.71	0.78	0.00	0.53	0.69	0.15	0.89	0.28	0.00	0.81	0.10	0.83	0.38	0.43	0.10	7.69
Guinea grass	0.04	0.60	0.49	0.94	0.92	0.27	0.02	0.87	0.50	0.61	0.11	0.89	0.67	0.60	0.93	0.48	8.42
Soil sample	0.80	0.99	0.34	0.89	0.08	0.62	0.77	0.91	0.32	0.85	0.40	0.12	0.01	0.38	0.66	0.68	8.80

*Choba*																	
Elephant grass	0.31	0.63	0.22	0.28	0.31	0.71	0.60	0.32	0.27	0.53	0.28	0.51	0.46	0.28	0.97	0.28	6.96
Maize	0.54	0.58	0.04	0.11	0.45	0.41	0.41	0.07	0.69	0.76	0.01	0.97	0.36	0.89	0.14	0.05	6.51
Guinea grass	0.48	0.23	0.83	0.50	0.37	0.98	0.54	0.64	0.74	0.12	0.04	0.86	0.20	0.47	0.18	0.16	7.31
Soil sample	0.83	0.26	0.73	0.59	0.64	0.56	0.67	0.38	0.08	0.14	0.42	0.20	0.32	0.56	0.72	0.30	7.42

*Khana*																	
Elephant grass	0.00	0.00	0.00	0.00	1.62	0.00	0.06	0.49	4.22	0.05	0.13	0.00	0.00	0.00	0.00	0.00	6.58
Maize	0.00	0.00	0.00	0.00	0.00	0.00	0.74	0.33	0.91	0.72	0.00	0.00	0.00	0.00	0.00	0.00	2.70
Guinea grass	1.14	11.9	1.88	0.38	1.66	0.09	0.52	1.35	0.61	0.39	0.76	0.71	11.1	0.00	0.00	0.00	32.5
Soil sample	0.00	0.00	0.00	0.00	0.00	0.00	2.82	0.00	0.00	0.00	0.00	3.36	0.15	0.00	0.00	0.00	6.33

*Yenagoa*																	
Elephant grass	0.18	0.51	0.15	0.78	0.54	0.60	0.70	0.62	0.78	0.82	0.55	0.02	0.84	0.29	0.52	0.18	8.06
Maize	0.85	0.65	0.32	0.79	0.37	0.28	0.47	0.92	0.66	0.50	0.81	0.76	0.02	0.91	0.59	0.84	9.71
Guinea grass	0.32	0.82	0.68	0.00	0.34	0.53	0.24	0.46	0.08	0.65	0.95	0.91	0.48	0.43	0.18	0.12	7.17
Soil sample	0.59	0.89	0.67	0.13	0.23	0.00	0.29	0.10	0.36	0.17	0.71	0.97	0.85	0.64	0.72	0.07	7.39

*Eleme*																	
Elephant grass	0.14	0.24	0.28	0.98	0.74	0.21	0.89	0.34	0.43	0.78	0.78	0.62	0.04	0.19	0.85	0.72	8.22
Maize	0.47	0.89	0.23	0.85	0.07	0.43	0.75	0.87	0.09	0.51	0.73	0.96	0.62	0.81	0.78	0.50	9.52
Guinea grass	0.86	0.37	0.51	0.26	0.30	0.23	0.02	0.14	0.55	0.29	0.24	0.70	0.84	0.89	0.32	0.44	6.96
Soil sample	0.19	0.58	0.33	0.18	0.53	0.52	0.36	0.16	0.25	0.92	0.13	0.39	0.83	0.49	0.10	0.83	6.79

*Trans-Amadi*																	
Elephant grass	0.25	0.55	0.91	0.69	0.02	0.30	0.77	0.17	0.16	0.65	0.21	0.19	0.40	0.96	0.55	0.45	7.21
Maize	0.65	0.94	0.61	0.08	0.47	0.27	0.74	0.87	0.08	0.06	0.46	0.21	0.42	0.44	0.06	0.72	7.07
Guinea grass	0.09	0.63	0.52	0.16	0.85	0.11	0.53	0.74	0.58	0.85	0.59	0.69	0.54	0.67	0.84	0.02	8.42
Soil sample	0.82	0.16	0.71	0.23	0.51	0.24	0.77	0.99	0.27	0.03	0.92	0.11	0.46	0.89	0.74	0.13	7.97

**Table 2:** PAH concentration in soil samples from different countries, *μ*g·kg^−1^ dry weight.

Soils	Region/area	No. of PAH	Range	Mean	Reference
Forest soil	North Bavaria	20		666	Krauss et al. [47]
Switzerland	Arabic soils	16	60–145	66	Bucheli et al. [48]
Agricultural soils	Korea	16		158	Nam et al. [49]
Agricultural soils	Shantou China	16	22–1256	318	Hoa et al. [50]
Urban agricultural soil	Bayreuth	20	280–2200	640	Krauss and Wilcke [51]
Urban soils	New Orleans	16		3700	Mielke et al. [52]
Indira Gandhi International Airport soil	India	12	2394–7529	4430 ± 1450	Ray et al. [53]
Military airfield soils	Poland	16	249–5657	—	Baran et al. [54]
West Macedonia lignite-fired power plants	Greece	16	55.2–495	—	Stalikas et al. [55]
Linz industrial area	Austria	18		1450	Weiss et al. [56]
Tianjin industrial area	China	16	818.2 ± 796.2	—	Wang et al. [57]
Novi Sad oil refinery (after Kosovo War)	Serbia and Montenegro	16	47870	—	Škrbic´ and Miljevic´ [58]
Kohtla-Jarve oil shale thermal treatment, industry, power station, and traffic	Estonia	18	12390 ± 9810	—	Trapido [59]
50 m from an oil refinery (Zelzatz)	Belgium	7	300,000	—	Bakker et al. [60]
1.3–4.2 km from an oil refinery	Belgium	7	3000–14000	—	Bakker et al. [60]
Coal mine soil	Huaibei and Huainan China	16	130–3540	840	Wang et al. [61]
Industrial areas soil	Yangtze River Delta, China	16	189.5–1070.4	471.30	Wang et al. [16]
Soils around a chemical plant	Shanxi, China	16	3870–76000.0	12600	Jiao et al. [62]
Forest fires soil	South Korea	16	—	1.570	Kim et al. [63]
Rural, urban, and industrial soils	Estonia	16	50−22	—	Trapido [59]
Urban soil	USA (Miami, Florida)	16	251–2364	—	Banger et al. [64]
Mangrove fresh soil	Nigeria (Lagos)	16	65.5–188.0	—	Sojinu et al. [65]
Floodplain soil	Nigeria (Niger Delta)	16	812–10700	—	Tesi et al. [66]
Urban soils	Nigeria (Niger Delta)	16	182–433	—	Abbas and Brack [67]
Soil vicinity oil installation	Nigeria (Niger Delta)	16	24–120	—	Sojinu et al. [68]
Urban soils	Nigeria (Niger Delta)	16	188–684	—	Iwegbue et al. [69]
Agricultural soil	Nigeria (Choba, Niger Delta)	16	—	7417	This study
Agricultural soil	Nigeria (Eleme, Niger Delta)	16	—	6787	This study
Agricultural soil	Nigeria (Khana, Niger Delta)	16	—	6325	This study
Agricultural soil	Nigeria (Trans-Amadi, Niger Delta)	16	—	7972	This study
Agricultural soil	Nigeria (Uyo, Niger Delta)	16	—	8799	This study
Agricultural soil	Nigeria (Yenagoa, Niger Delta)	16	—	7394	This study

**Table 3:** Percentage composition of molecular weight distribution of PAHs in vegetation and soil from different locations in the Niger Delta.

Samples	LMW	MMW	HMW
*Uyo*			
Elephant grass	33.3	32.7	34.0
Maize	36.7	17.1	46.0
Guinea grass	38.5	23.7	37.7
Soil sample	42.1	32.3	25.7

*Choba*			
Elephant grass	35.5	24.6	39.9
Maize	33.3	29.6	37.1
Guinea grass	46.3	27.7	26.0
Soil sample	48.0	17.2	34.1

*Khana*			
Elephant grass	24.6	73.4	2.01
Maize	0.22	99.6	0.22
Guinea grass	52.2	7.88	39.9
Soil sample	0.09	44.6	55.3

*Yenagoa*			
Elephant grass	34.1	36.1	29.8
Maize	33.3	26.4	40.3
Guinea grass	37.5	19.8	42.7
Soil sample	34.1	12.4	53.5

*Eleme*			
Elephant grass	31.5	29.7	38.8
Maize	30.8	23.3	45.9
Guinea grass	36.3	14.5	49.2
Soil sample	34.4	24.9	40.8

*Trans-Amadi*			
Elephant grass	28.2	23.9	47.9
Maize	42.8	24.9	32.5
Guinea grass	27.1	32.1	39.9
Soil sample	33.6	25.9	40.5

**Table 4:** Molecular ratio of select PAHs for identification of possible sources in soils and plants.

PAH ratios	Values	Possible sources	Uyo		Choba		Khana		Yenagoa		Eleme		Trans-Amadi	
			SS	PS	SS	PS	SS	PS	SS	PS	SS	PS	SS	PS
Fle/(Fle + Pyr)	<0.5	Petrol emissions	0.49 (0.87–1.78)	0.32 (1.29–3.97)	0.60 (0.59–0.97)	0.46 (0.89–1.91)	0.5 (0.001–0.002)	0.15 (0.38–2.55)	0.58 (0.12–0.21)	0.44 (1.56–3.55)	0.32 (0.17–0.54)	0.60 (2.09–3.44)	0.19 (0.23–1.22)	0.34 (0.93–2.70)
	>0.5	Diesel emissions												

Ant/(Ant + Phe)	<0.1	Petrogenic	0.88 (0.61–0.70)	0.48 (1.69–3.49)	0.46 (0.56–1.20)	0.64 (2.09–3.23)	0.5 (0.001–0.002)	0.02 (0.09–3.37)	0.01 (0.002–0.23)	0.52 (1.39–2.64)	0.49 (0.52–1.05)	0.44 (0.87–1.96)	0.31 (0.23–0.75)	0.33 (0.68–2.01)
	>0.1	Petroleum, biomass comb												

Flt/(Flt + Pyr)	<0.4	Petrogenic	0.45 (0.77–1.67)	0.21 (0.74–3.42)	0.63 (0.67–1.05)	0.60 (1.54–2.57)	0.10 (2.82-2.82)	0.37 (1.31–3.48)	0.74 (0.28–0.38)	0.41 (1.40–3.39)	0.69 (0.36–0.52)	0.55 (1.66–3.01)	0.43 (0.77–1.76)	0.53 (2.01–3.79)
	0.4–0.5	Fossil fuel combustion												
	>0.5	Biomass, coal comb												

BaA/(BaA + Cry)	<0.2	Petrogenic	0.27 (0.32–1.17)	0.51 (1.63–3.16)	0.36 (0.08–0.23)	0.54 (1.70–3.09)	0.5 (0.001–0.002)	0.83 (5.73–6.89)	0.68 (0.36–0.53)	0.43 (1.52–3.49)	0.20 (0.25–1.17	0.40 (1.07–2.64)	0.89 (0.27–0.30)	0.34 (0.83–2.38)
	0.2–0.35	Petroleum comb												
	>0.35	Biomass, coal comb												

BbF/BkF	0.92	Wood comb	0.30 (0.12–0.4)	0.47 (1.32–2.81)	0.5 (0.2–0.42)	0.13 (0.32–2.34)	0.00 (0.001–3.35)	0.8 (0.71–0.89)	0.73 (0.71–0.97)	0.73 (1.68–2.31)	0.33 (0.13–0.39)	0.76 (1.74–2.28)	0.12 (0.11–0.92)	1.16 (1.27 − 1.09)
	1.07	Diesel engine												
	1.3	Vehicular emission												
	3.7	Coal combustion												

BaP/BghiP	0.3–0.78	Vehicular emission	0.01 (0.01–0.66)	0.85 (1.88 − 1.61)	0.44 (0.32–0.72)	0.78 (1.02–1.29)	0.01 (0.001–0.14)	0.00 (0.003–11.13)	0.84 (0.72–0.85)	0.96 (1.28–1.33)	0.12 (0.10–0.83)	0.76 (1.49–1.94)	0.62 (0.46–0.74)	0.92 (1.34–1.45)
	0.9–6.6	Coal comb												

BaP/(BaP + Cry)	<0.49	Gasoline	0.009 (0.01–0.86)	0.55 (1.89–3.42)	0.69 (0.32–0.47)	0.42 (1.02–2.41)	0.99 (0.14–0.15)	0.92 (11.34–12.30)	0.83 (0.85–1.02)	0.40 (1.33–3.31)	0.47 (0.82–1.75)	0.48 (1.49–3.06)	0.93 (0.46–0.49)	0.46 (1.34–2.89)
	>0.50 –0.73	Diesel engine												

Ind/(Ind + BghiP)	<0.2	Petrogenic	0.50 (0.68–1.35)	0.45 (1.30–2.91)	0.29 (0.30–1.02)	0.27 (0.49–1.78)	0.5 (0.001–0.002)	0.5 (0.003–0.006)	0.08 (0.07–0.79)	0.47 (1.14–2.41)	0.89 (0.82–0.92)	0.46 (1.65–3.59)	0.12 (0.11–0.85)	0.45 (1.19–2.64)
	0.2–0.5	Petroleum comb												
	>0.5	Biomass, coal comb												

SS = soil sample; PS = plant sample.

**Table 5:** Isomeric ratio and the total index of soil from different locations in the Niger Delta, Nigeria.

Compounds	Uyo	Choba	Khana	Yenagoa	Eleme	Trans-Amadi
Flt/(Flt + Pyr)	0.43	0.54	1.00	0.74	0.70	0.44
Ant/(Ant + Phen)	0.87	0.48	0.50	0.01	0.50	0.32
Phen/Ant	0.13	1.14	1.00	11.00	1.02	2.16
LMW/HMW	1.64	1.41	0.00	0.64	0.84	0.88
BaA/(BaA + Chry)	0.27	0.37	0.50	0.62	0.21	0.89
IndP/(IndP + BghiP)	0.51	0.29	0.50	0.09	0.89	0.13
Total index	12.4	8.69	10.1	5.531	9.52	8.96

**Table 6:** RQ_(NCs),_ RQ∑PAHs_(NCs),_ RQ_(MPCs),_ and RQ∑PAHs_(MPCs)_ values for PAH from different regions in the Niger Delta.

RQ_(NCs)_							RQ_(MPCs)_						
	Uyo	Choba	Khana	Yenagoa	Eleme	Trans-Amadi		Uyo	Choba	Khana	Yenagoa	Eleme	Trans-Amadi
Nap	796	829	1	594	195	823	Nap	7.96	8.29	0.01	5.94	1.95	8.23
Acy	998	263	1	894	580	162	Acy	9.98	2.63	0.01	8.94	5.80	1.62
Ace	335	730	1	672	331	706	Ace	3.35	7.30	0.01	6.72	3.31	7.06
Flu	875	589	1	125	177	234	Flu	8.75	5.89	0.01	1.25	1.77	2.34
Phe	79	640	1	234	529	513	Phe	0.79	6.40	0.01	2.34	5.29	5.13
Ant	61.7	56.4	0.1	0.20	51.9	23.7	Ant	6.17	0.56	0.001	0.002	0.52	0.24
Flt	766	667	—	288	364	772	Flt	7.66	6.67	28.2	2.88	3.64	7.72
Pyr	907	382	1	99	157	988	Pyr	9.07	3.82	0.01	0.99	1.57	9.88
BaA	3.19	8.30	0.01	3.63	2.45	2.72	Baa	0.03	0.08	0.00	0.04	0.03	0.03
Cry	84.9	14.4	0.1	16.9	92.2	3.3	Cry	8.49	0.14	0.00	0.17	0.92	0.03
BbF	4.02	4.20	0.01	7.08	1.30	9.19	BbF	0.04	0.04	0.00	0.07	0.01	0.09
BkF	1.19	2.03	33.7	9.66	3.92	1.13	BkF	0.01	0.02	0.34	0.10	0.04	0.01
BaP	0.01	0.32	0.15	0.85	0.83	0.46	BaP	0.00	0.00	0.00	0.01	0.01	0.01
DahA	0.38	0.57	0.001	0.64	0.49	0.88	DahA	0.04	0.01	0.00	0.06	0.01	0.01
BghiP	66.2	72.3	1	72.3	10.3	74.3	BghiP	0.66	0.73	0.01	0.72	0.10	0.74
Ind	68.3	29.9	1	6.9	82.5	10.8	Ind	0.68	0.03	0.01	0.07	0.83	0.11
RQ∑PAHs(NCs)	5,045.9	4,288.4	2,858.8	3,024.1	2,578.9	4,324.4	RQ∑PAHs(MPCs)	50.5	42.9	28.6	30.2	25.8	43.2

## Data Availability

The data used to support the findings of this study are included within the article.

## Additional Points

*Highlights*. (i) There is a widespread PAH pollution in the Niger Delta, Nigeria, (ii) Nap, Acy, Ace, Flu, Ant, Flt, and Pyr pose major ecological risk of PAHs in soils, (iii) PAHs from the Niger Delta emanated from pyrogenic processes and petrogenic sources, and (iv) some soil in the Niger Delta may require remediation.
